# Analysis of high-depth sequence data for studying viral diversity: a comparison of next generation sequencing platforms using Segminator II

**DOI:** 10.1186/1471-2105-13-47

**Published:** 2012-03-23

**Authors:** John Archer, Greg Baillie, Simon J Watson, Paul Kellam, Andrew Rambaut, David L Robertson

**Affiliations:** 1 Computational and Evolutionary Biology, Faculty of Life Sciences, University of Manchester, Manchester, UK; 2 Wellcome Trust Sanger Institute, Cambridge, UK; 3 UCL/MRC Centre for Medical Molecular Virology, Division of Infection and Immunity, University College London, London, UK; 4 Institute of Evolutionary Biology, University of Edinburgh, Edinburgh, UK; 5 Fogarty International Center, National Institutes of Health, Bethesda, MD, USA

## Abstract

**Background:**

Next generation sequencing provides detailed insight into the variation present within viral populations, introducing the possibility of treatment strategies that are both reactive and predictive. Current software tools, however, need to be scaled up to accommodate for high-depth viral data sets, which are often temporally or spatially linked. In addition, due to the development of novel sequencing platforms and chemistries, each with implicit strengths and weaknesses, it will be helpful for researchers to be able to routinely compare and combine data sets from different platforms/chemistries. In particular, error associated with a specific sequencing process must be quantified so that true biological variation may be identified.

**Results:**

Segminator II was developed to allow for the efficient comparison of data sets derived from different sources. We demonstrate its usage by comparing large data sets from 12 influenza H1N1 samples sequenced on both the 454 Life Sciences and Illumina platforms, permitting quantification of platform error. For mismatches median error rates at 0.10 and 0.12%, respectively, suggested that both platforms performed similarly. For insertions and deletions median error rates within the 454 data (at 0.3 and 0.2%, respectively) were significantly higher than those within the Illumina data (0.004 and 0.006%, respectively). In agreement with previous observations these higher rates were strongly associated with homopolymeric stretches on the 454 platform. Outside of such regions both platforms had similar indel error profiles. Additionally, we apply our software to the identification of low frequency variants.

**Conclusion:**

We have demonstrated, using Segminator II, that it is possible to distinguish platform specific error from biological variation using data derived from two different platforms. We have used this approach to quantify the amount of error present within the 454 and Illumina platforms in relation to genomic location as well as location on the read. Given that next generation data is increasingly important in the analysis of drug-resistance and vaccine trials, this software will be useful to the pathogen research community. A zip file containing the source code and jar file is freely available for download from http://www.bioinf.manchester.ac.uk/segminator/.

## Background

Sequencing platforms such as the 454 Life Sciences GS-FLX [1] and Illumina [2] are providing a previously unprecedented insight into the extent of pathogen variation [3-7]. This is due to the depth of coverage that can be obtained across individual genes or genomes [8], as well as the ability to analyze large numbers of samples simultaneously [9,10]. Within chronic viral infections, such as HIV-1 and HCV, the study of variation is important as it has been directly associated with both disease progression and the outcome of treatment [11-14]. These sequencing platforms have the potential to accurately quantify the variation within viral populations [15,16], including those sampled through time [3,17], and from differing compartments within the host [18], thus, providing a more complete picture of viral evolution. This has applications for the development of treatment strategies that are both reactive and predictive. Reactive in that the information derived from individual hosts can be incorporated into the optimization of current drug treatment regimes [14,19-21]. Predictive in that the likelihood of the emergence of resistant variants can be calculated and incorporated into strategies such as the algorithmic design of therapeutic vaccines [22,23].

However, prior to the practical and routine application of current sequencing technologies to the characterization of patterns of variation, a number of non-trivial challenges need to be overcome [24]. Primarily this involves the detection of error introduced during sequencing [25,26], and the separation of this from real genetic variation. This issue is particularly acute for RNA viruses within an individual host for which the degree of genetic variation may be on the same order as the error rate. The extent and nature of sequencing error varies between platforms [27]. For example, the 454 technology utilizes a sequencing by-synthesis method during which the incorporation of cyclically delivered nucleotides into the growing DNA strand, via pyrophosphate liberation, is measured [28]. In regions where the complement strand contains a homopolymeric stretch, the strength of the signal is proportional to the number of bases incorporated. Ambiguities in signal intensity are most frequently manifested as under- and over-calls on the length of these stretches [29]. An overall error rate of about 1% has been observed [7,25] which has been partitioned into insertion (0.7%), deletion (0.2%) and mismatch error (0.1%). Unlike the 454 platform, sequences generated on the Illumina platform are extended one base at a time [28], thus, under- and over-calls are uncommon. Mismatch error, however, has been observed [26,27], and, because of the high coverage that is achievable, the quantification of the rate at which this error occurs is particularly relevant when attempting to identify low frequency genetic variants.

The development of an accurate, dynamic picture of viral evolution within the host has also been hampered by the logistics of data management. The many mapping, assembly and analysis programs available [30,31] primarily have the goal of obtaining an accurate estimate of a genomic sequence or detecting variation at the allelic level. Other commercially available packages such as Geneius [32] and CLC Genomics Workbench http://www.clcbio.com/, that do offer tools to analyze sequence variability, have limited capacity to account for temporally sampled viral data. At the time of writing no freely available, generally applicable software exists that addresses the task of detecting and characterizing the high levels of genetic variation encountered within rapidly evolving viral populations using data that has either been temporally or compartmentally sampled. Prior to our current work we presented a framework, for the mapping of the short sequence segments (reads) generated on the 454 platform, which was applied to the detection of clusters of low frequency drug resistant forms [3]. Here we extend this framework so that it (i) is applicable to viral data generated on the Illumina platform (and other sequencing platforms), (ii) outputs a range of metrics for characterizing variability including base, indel and codon frequencies, coverage and quality scores and (iii) allows for multiple data sets to be managed within a project permitting comparative analysis. To demonstrate the usefulness of our software we assess platform-induced variation present within reads derived from 12 Influenza A H1N1/09 infected individuals. For each individual, because the same sample was used for read amplification on both the 454 and Illumina platforms, any variation associated solely with a single platform can be considered as potential sequencing error. H1N1 genomes are particularly useful for characterizing insertion and deletion error that has been introduced by the sequencing platform because indels are rare in this data [5].

## Implementation

**Figure 1:**
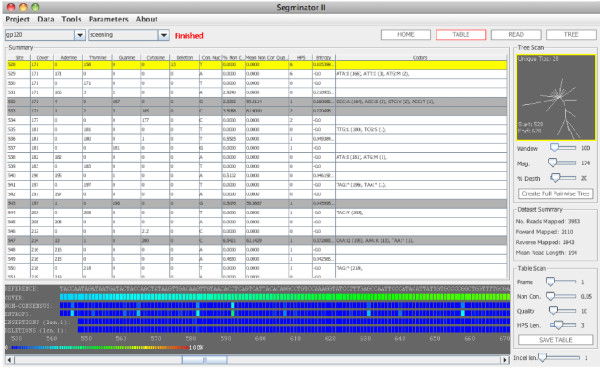
**Segminator II user interface**. The table in the center of the GUI displays information that is available after read mapping and alignment. Above the table are the dropdown boxes that allow navigation between projects and data sets. Below the table heatmaps showing cover, entropy, and insertion, deletions and non-consensus frequencies are located. On the right hand side the "Treedar" feature which dynamically generates a neighbor joining tree as the user scrolls along the genome is located. Below this are data set summary panel and the table scan panel, the latter which can be used to query sites based on variation, quality and homopolymeric stretch size (grey rows). On the top right buttons to change the display mode to the read view or tree view are observed.

**Figure 2:**
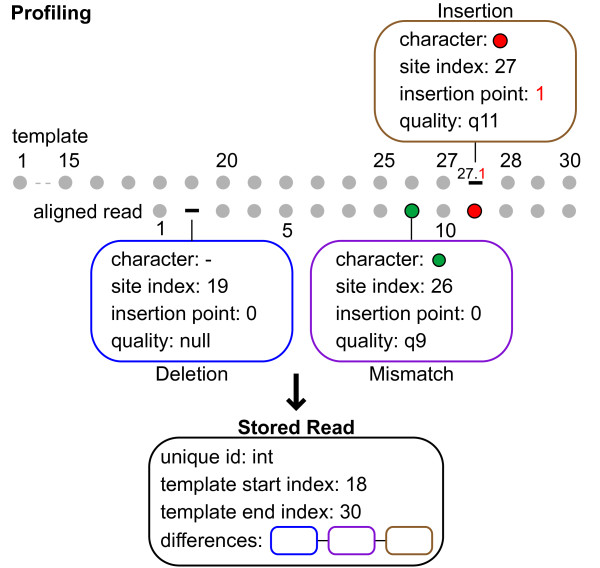
**Read storage**. Profiling of a single read in relation to the template (top) and subsequent data structure (bottom) used to store the polymorphic information within the assembly. Blue, purple and brown boxes indicate a deletion, mismatch and insertion, respectively.

Implemented in Java the software, Segminator II, along with the source code, is available from http://www.bioinf.manchester.ac.uk/segminator/. A graphical user interface (Figure 1) resides on top of an underlying data management framework providing convenient access to the main features of the software. Components of the framework can be incorporated within automated bioinformatics pipelines through direct use of the core package (available with the source code). Within the framework individual reads are stored post-mapping as lists of polymorphisms in relation to a user-defined reference sequence (Figure 2). As a consequence the majority of characters that are identical to the template, representing redundant information, do not need to be stored. Selection of a reference sequence that is proximal to the data set is, therefore, important not only for improved mapping [3], but also for the reduction in the quantity of information that needs to be stored. The storage of polymorphic characters in this manner allows for the efficient extraction of data from the assembly. For example, reads themselves spanning a particular location of the template can be quickly reconstructed either (i) with all insertions in relation to the template removed, thus, maintaining site compatibility between reads or (ii) in its original format, where the read start location is maintained but inter-read compatibility between individual sites is not guaranteed. An immediate application of the former is the 'Treedar' feature of the user interface (Figure 1) which uses pairwise distances between reads that have been aligned to the template in order to generate a rough, but dynamically generated, phylogenetic tree as the user scrolls along the virus genome. This tree can be used to rapidly identify regions of the genome with divergent portions of sequence space prior to the localized application of full phylogenetic reconstruction techniques. Additionally access to information pertaining to the classification of variation at individual sites (or codons), along with associated quality scores, is made possible without the need to reconstruct complete alignments.

**Figure 3:**
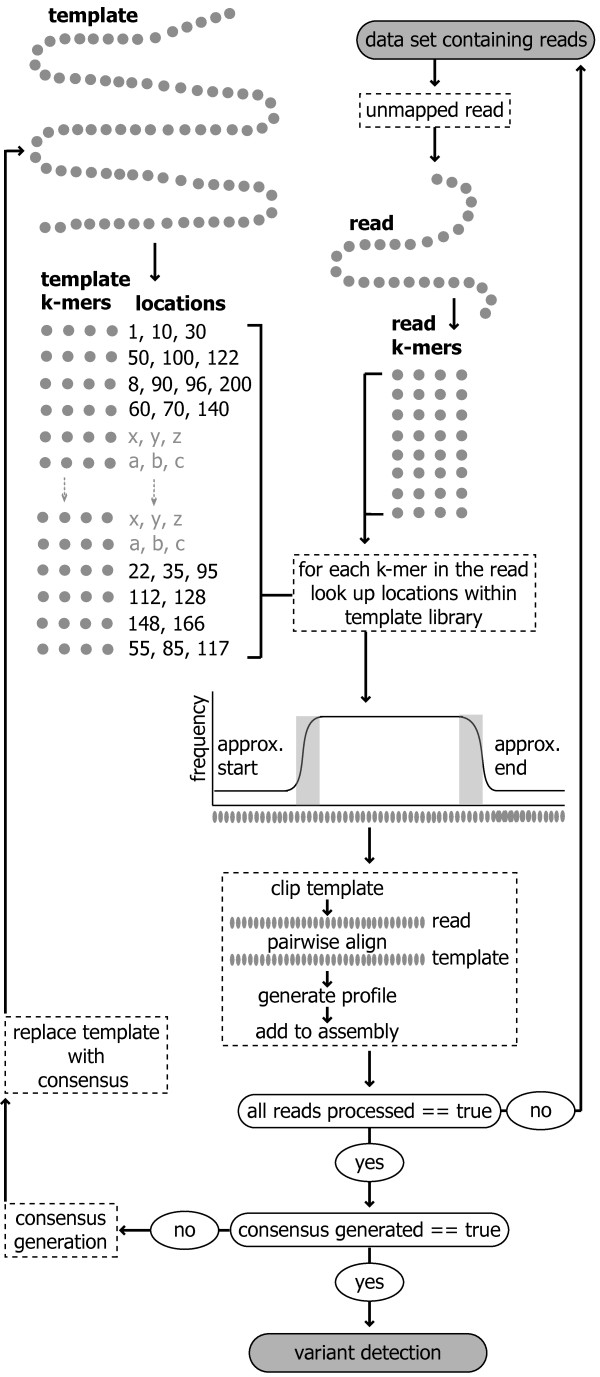
**The data analysis pipeline**. The preprocessing of the template sequence prior to read mapping is outlined (top left). The fragments titled "k-mers", represented by gray dots, are all the unique words within the template sequence. These are stored along with their corresponding locations. On the top right all k-mers of equal length, extracted from the read, are shown. The plot indicates the frequency of k-mer matches across the template sequence for a single read. Grey boxes indicate processing events that take place within the framework. The bottom left indicates the beginning of a second iteration of this pipeline after a data specific template has been generated. The condition for this second iteration to begin is illustrated in the white circle.

Prior to read storage and variant detection individual reads in fastq format, are required to be mapped to the template sequence. This is performed using a combination of k-mer matching and pairwise alignment (Figure 3). Through the user interface multiple data sets may be linked to the same template sequence. Setting up a project with its associated template sequence does this as subsequent data sets added to the project are then mapped to the associated template. If the user is concerned about failing to map reads because of divergence from the template, which is especially relevant to rapidly evolving viral genomes such as HIV-1 [3], there is a parameter (under the Parameters- > Miscellaneous menu option) that when set to true, will take a consensus at each site of the template after the first mapping and then remap reads to this consensus sequence. A full description of the features of the user interface, as well as the parameters used, is available on the website. To demonstrate the usage of our framework we have applied it in a case study to 12 H1N1 data sets derived from samples sequenced on both the 454 and Illumina platforms.

### Case study: data sets

Nasal swabs were taken from individuals within the UK presenting with pandemic H1N1/2009. Virus genome RNA was extracted from these swabs using standard methods. Influenza genomes were RT-PCR amplified using the method based on [33]. Reactions were performed in a volume of 50 μl under an overlay of 20 μl Vapor-Lock (Qiagen), and contained 5.0 μl of RNA isolated from clinical material, and final concentrations of 1× SuperScript^® ^III One-Step RT-PCR reaction buffer, 0.5 μM each primer and 1.0 μl SuperScript^® ^III RT/Platinum^® ^Taq High Fidelity Enzyme Mix. Thermal cycling conditions were: reverse transcription at 42°C for 15 minutes, 55°C for 15 minutes, 60°C for 5 minutes; initial denaturation/enzyme activation of 94°C for 2 minutes; five cycles of 94°C for 30 seconds, 45°C for 30 seconds, slow ramp (0.5°C/sec) to 68°C, 68°C for 3 minutes; 30 cycles 94°C for 30 seconds, 57°C for 30 seconds, 68°C for 3 minutes; and final extension of 68°C for 5 min. RT-PCR products were used to generated multiplex identifier (MID) tagged libraries for 454 (454 Life Sciences, Branford, CT) or Illumina (Illumina Inc., San Diego, CA) sequencing, according to the manufacturers' instructions. 454 sequencing was performed on the Genome Sequencer FLX system. Illumina sequencing was performed on the Genome Analyzer I system with 54 bp paired-end reads. In total there were 24 data sets, 12 from each platform. The number of reads available for each data set prior to mapping ranged from 832,117 to 2,827,392 for the Illumina platform, and from 4,569 to 14,758 for the 454 platform (Table 1). These are available in fastq format from our website.

**Table 1:** Sample ID's and read numbers before and after mapping

	Illumina		454 Life Sciences	
**Sample ID**	**No. of reads**	**No. of reads mapped (%)**	**No. of reads**	**No. of reads mapped (%)**

**511**	1160281	875620 (75)	4569	4082 (89)

**512**	2266246	1586144 (70)	5173	4286 (83)

**513**	2606959	1794027 (69)	9679	8972 (93)

**533**	1751178	1264611 (72)	6974	6339 (91)

**534**	1428288	1015404 (71)	7642	7093 (93)

**535**	832117	655689 (79)	10176	9444 (93)

**538**	1387955	1115917 (80)	6690	6282 (94)

**540**	2827392	2136159 (76)	5419	4985 (92)

**541**	1574181	1276311 (81)	5151	4835 (94)

**578**	2870922	2130089 (74)	17586	16307 (93)

**579**	1664978	1208913 (73)	14758	13657 (93)

**580**	1854234	1378426 (74)	14124	12990 (92)

Unmapped and mapped reads

### Case study: template creation, mapping and alignment

To construct a reference sequence, for each genomic segment of the H1N1 genome, a sample of data-all available full length US sequences-were downloaded from GenBank http://www.ncbi.nlm.nih.gov/genbank/, aligned using Muscle [34] and a consensus sequence generated. The number of sequences included for each gene was: PB2: 1401, PB1: 1397, PA: 1387, HA: 1578, NP: 1461, NA: 1586, MP: 1490 and NS: 1409. Consensus sequences from each were concatenated together to produce a genome length template of length 13,284 nucleotides. At each concatenation point, a string of Ns was incorporated in order to separate the segments for visualization purposes; these were ignored in the read mapping. For each of the 12 samples, reads from both platforms were separately mapped to the template using our framework (Figure 2), following which a data set specific consensus sequence was generated by maintaining the most frequent residue present at each site. Reads were then remapped and aligned to the consensus sequence, which resulted in the final assemblies used in downstream analysis. The parameters for the k-mer matching step were k-mer length, k-mer density (minimum number of k-mer hits before a location is considered) and k-mer skip (rather than search every k-mer within the read every i^th ^k-mer is used, where i is the skip parameter); the values set for these were 8, 2 and 2, respectively. Note, for conserved data the speed of the mapping can be increased using the skip parameter as searching every k-mer to find the approximate location of a read leads to redundancy. For the pairwise alignment step the parameters were: match, gap open and gap extension scores as well as the value for transversions and transitions with the values: 1, -2, -0.5, 2 and 1, respectively.

### Case study: results

**Figure 4:**
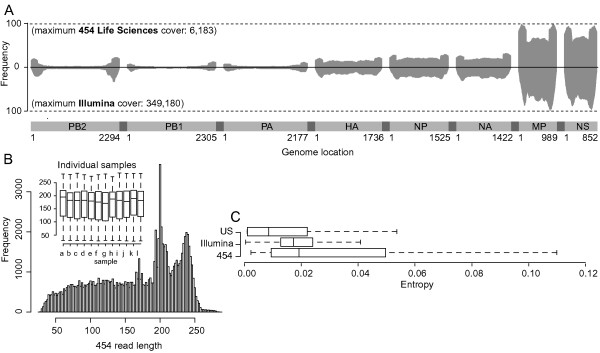
**Initial mapping and data characterization**. **(A) **Normalized read coverage across the eight genomic segments of the H1N1 genome (data from each sample has been combined). Coverage obtained from the 454 platform is displayed above the x-axis while the coverage obtained on the Illumina platform is mirrored below. In each case the maximum value is depicted in brackets. Genomic segments are shown along the bottom of the plot. **(B) **Read length variation across the 454 data following sample pooling. The inset box and whisker plot shows the read lengths within each individual sample labeled a to l and corresponding to samples 511 to 580, respectively (Table 1). **(C) **Entropy present within data obtained from the 454 and Illumina platforms as well as that present within in a sample of H1N1 data from GenBank.

The number of successfully mapped reads from each sample is presented in Table 1. For each data set, following pooling of the data by platform, genomic coverage was calculated (Figure 4A). The median length of the reads within the combined 454 data sets was 182 nucleotides (Figure 4B), which varied only slightly within individual data sets (Figure 4B, inset). Reads from the Illumina platform were all of length 54 nucleotides. To characterize the general diversity present within our 12 samples the per-site entropy was calculated across data pooled according to platform using the standard Shannon entropy measure. For comparison, per-site entropy was also calculated within the sample of GenBank data used during the generation of the reference sequences. For both platforms the entropy present (454 median: 0.0192; Illumina median: 0.0172) was observed to be higher than that within the database data (median: 0.0085) (Figure 4C), thus, highlighting the need to characterize platform dependent variation within the read data. Note, the same pattern of mutations was seen when samples were analysed individually.

**Figure 5:**
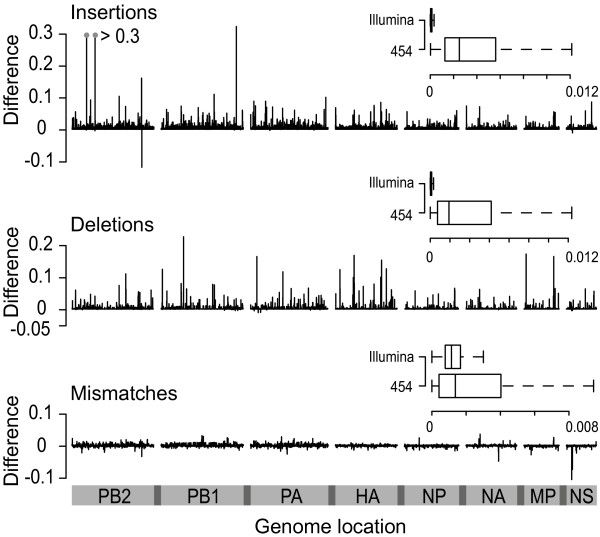
**Cross platform differences**. Subtraction of the per-site ratios obtained from each platform. Genomic segments are depicted along the bottom. The inset box and whisker plots represent the absolute values categorized according to their original polarity.

**Figure 6:**
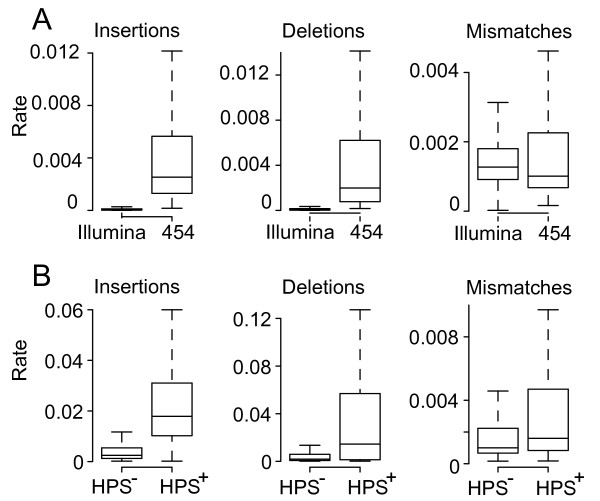
**Cross platform rate variation**. **(A) **Ratios between variant and consensus frequencies for insertions, deletions and mismatches, respectively, for each platform as compared to the consensus sequence. **(B) **Ratios between variant and consensus frequency states observed on the 454 platform within hps^+ ^and hps^- ^regions.

The ratio between the frequency of minority variants and the population consensus at each genomic site was calculated using data sets pooled according to platform. Here pooling was performed, as we were interested in characterizing differences occurring between platforms, not the explicit detection of variants within each sample. In order to identify platform-induced variation these ratios, after normalization for differences in coverage, were subtracted at each genomic site (Figure 5). Because the same samples had been used for sequencing on both platforms, differences that deviate from zero are indicative of platform specific variation, with positive values indicating variation unique to the 454 platform and *vice versa*. When ratios obtained from each platform are compared, the extent of platform dependent variation is highlighted (Figure 6A). The median for insertions and deletions within the data generated on the 454 platform, at 0.0025 and 0.0019, respectively, were significantly higher (p < 0.001, Wilcox rank sum test) than those from insertions and deletions within the data generated on the Illumina platform (0.00004 and 0.00006, respectively). For mismatches, the median rate obtained on the 454 platform, at 0.0010, was very similar to that observed with the Illumina data (0.0012). When sites are divided into two categories: (i) those that are part of a homopolymeric stretch of length three or more (hps^+^) and (ii) those that are outside of these regions (hps^-^), the 454 platform shows significantly higher variation occurring within the hps^+ ^category (Figure 6B). For insertions, deletions and mismatches the median within the hps^+ ^sites were 0.0179, 0.0145 and 0.0016, respectively, while outside of these regions the medians were 0.0025, 0.0019 and 0.001, respectively. Despite the far higher coverage, data from the Illumina platform contained very few insertions and deletions, and no difference between hps^+ ^and hps^- ^was observed.

**Figure 7:**
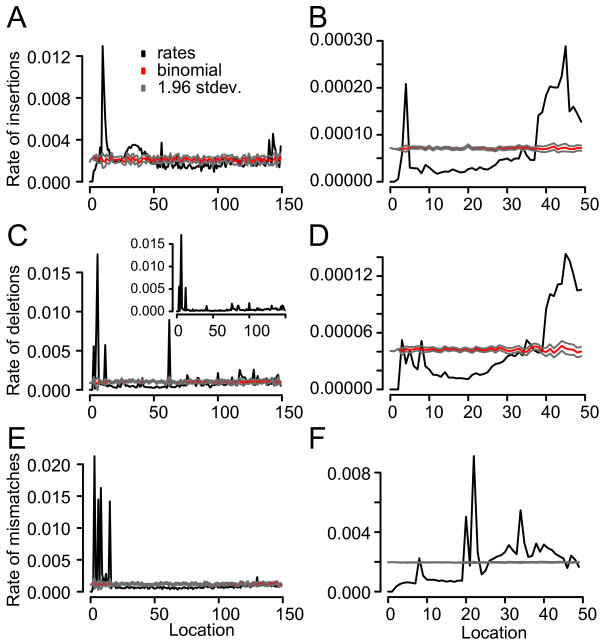
**Read location versus error**. Rates of insertions, deletions and mismatches across all template sites in relation to position on read for both the 454 **(A, C and E)**, respectively, and Illumina **(B, D and F) **platforms, respectively. The inset in C shows the rate of deletions across hps^- ^only sites on the template in relation to position on the read. In A and B the binomial expectation is shown (red) along with ± 1.96 standard deviations.

On mapping change from a consensus to a minority variant at sites on the template to locations on the underlying reads it was observed that within the 454 data, with the exception of the very start of the reads, there is a relatively uniform distribution across the read length, following a random expectation (Figure 7A, C and 7D). For deletions the single peak, at location 62, (Figure 7C) was attributed to a homopolymeric run of five cytosine's at position 62 of the MP segment and another of four adenine's at the same position of the PA segment. When sites on the template were limited to those that were outside of hps^+ ^regions of length four or more this peak was removed (Figure 7C, inset). Given that genome defined hps^+ ^regions explains the majority of platform-induced variation this uniform trend is not unusual. If this trend was not present it would suggest that read location plays a significant role in the introduction of variation, but, after taking the hps^+ ^regions into account, there is little variation left to explain. For the Illumina data there is a non-linear relationship between read location and observed variability (Figure 7B, D and 7F). Here however it should be noted that for insertions and deletions the overall rates are two orders of magnitude less than in their 454 counterparts, again, reflecting the robustness of the Illumina platform to the introduction of erroneous indels.

**Figure 8:**
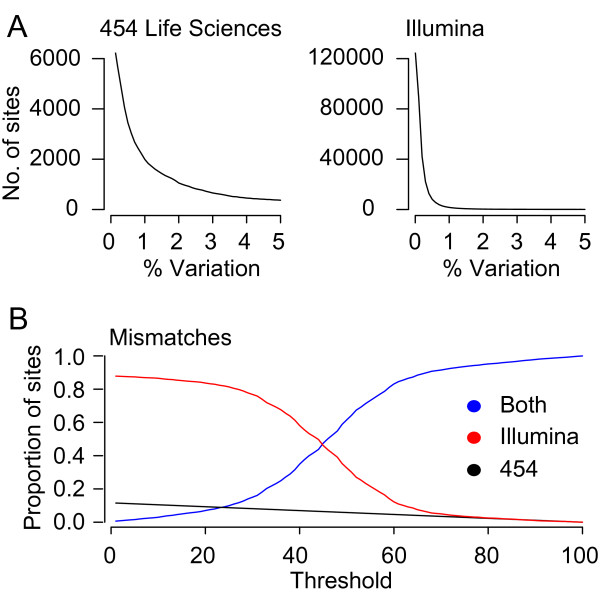
**Low level variability between both platforms**: **(A) **Number of variable sites containing non-consensus nucleotides at the level above that indicated by the x-axis. Sites were counted across each sample independently for both the 454 (left) and Illumina (right) platforms. **(B) **The proportion of sites displaying variation that are in agreement between platforms (blue); threshold levels (based on percentiles across the distribution of rate differences) are depicted on the x-axis. Red and black indicate variable sites that are present on within data from single platform only (see key).

The proportion of nucleotide sites across each patient that contain differences to the consensus was calculated (Figure 8A). Although the underlying rates for mismatches are similar between both platforms the Illumina data contains far more sites harboring low-level variation. This is a result of the characteristically much higher levels of coverage obtained from this platform. The proportion of sites across the template that contained variability unique to one of the platforms was then plotted against a range of threshold values that defined the extent of variation allowed between platforms before a site was considered to be in disagreement (Figure 8B). Threshold values were based on percentiles across the distribution of rate differences (Figure 5). For example, at the threshold value of 30 any values between 0 plus or minus the 30^th ^percentile value of rate differences was considered to be in agreement, and any value outside of this range was not in agreement. Intuitively as the threshold is increased the proportion of sites in agreement between both platforms is observed to increase. For the 454 data at low threshold levels, the proportion of sites harboring mismatches not confirmed on the Illumina platform, at 0.15, is low. However, on the Illumina platform, at the same low threshold levels, the proportion of sites harboring low-level variation, not confirmed on the 454 platform, is 0.85. It is only at threshold levels above the 60^th ^percentile that both platforms start to agree with each other consistently.

**Figure 9:**
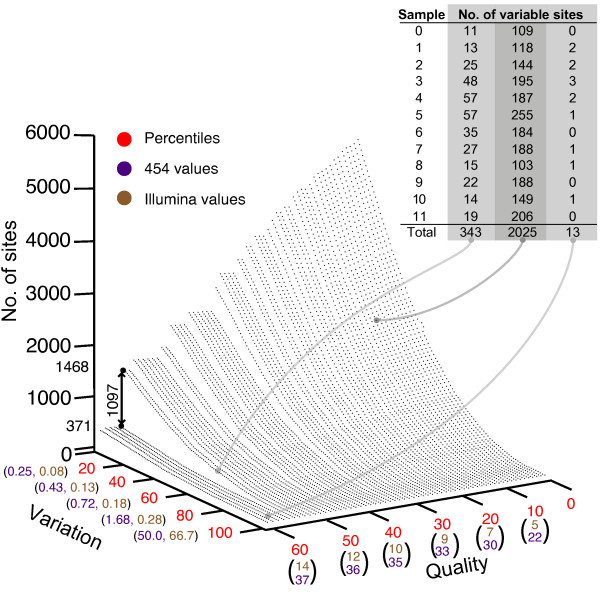
**Mismatches confirmed on both platforms**: Number of variable sites confirmed, within each sample independently, on both platforms at differing quality and variation thresholds. The inset table displays the number of variable sites associated with each sample for three points associated with three different parameter quadruples (light grey dots). The double-sided arrow indicates the drop in variable sites identified between the 55th and 56th quality score percentiles.

**Figure 10:**
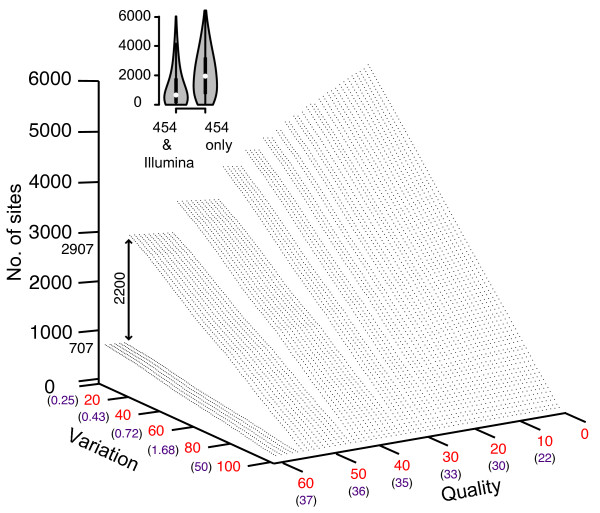
**Mismatches confirmed on the 454 platform**. Number of variable sites confirmed, within each sample independently, on the 454 platform at differing quality and variation thresholds. The inset violin plot depicts the differences between the number of variable sites confirmed on both platforms (Figure 9) to those confirmed using just the 454 platform. The double-sided arrow indicates the drop in variable sites identified between the 55^th ^and 56^th ^quality score percentiles.

Both frequency and base quality are usually used as an indication of the reliability of the observed variation. To visualize the relationship between (i) the percent of non-consensus nucleotides at a site, (ii) the quality of nucleotide calls, and (iii) the number of sites verified within each individual sample on both platforms, percentiles were used to select incrementing minimum threshold values for quality and variation. For each parameter set, the number of sites across all patients with variation and quality greater than or equal to the values used for both platforms could be identified (Figure 9). For example, if the i^th ^site within the data from an individual patient obtained from the 454 platform had both variation and quality above the selected percentile value for that platform, but the corresponding site within the Illumina data fell below one or both of the Illumina percentile values, the site would not be counted. Alternatively if the i^th ^site on the Illumina platform also had variation and quality greater than or equal to the Illumina selected percentile values then the site would be counted. Thus, this provides a method of identifying variable sites containing a minimum level of variation, associated with a minimum quality, that has been cross-validated on both platforms. Three examples of such site identification are depicted (Figure 9, inset). An interesting feature of this topology is between the 55^th ^and 56^th ^quality score percentile, there is a marked decrease in the number of sites identified across all non-consensus values. On the 454 platform the quality values at these percentiles was 36 and 37, respectively, while on the Illumina platform the corresponding quality values were 13 and 14. If variable sites are not validated using the Illumina platform the number of sites identified within each of the 12 samples is consistently higher (Figure 10). Across all 6000 parameter pairs queried the median number of variable sites identified is 1947, just under three times higher than when cross-platform validation was performed (median: 662) (Figure 10, inset). If data from the Illumina platform was used without validation from the 454 platform, the number of variable sites obtained is consistently higher than both previous searches since the number of sites containing low-level variation exceeds that within the 454 data.

## Discussion

Here we have developed a framework for the comparison of next generation data derived from multiple sources. We have applied the framework to the comparison of platform dependent error rates present within data generated on both the 454 Life Sciences and Illumina platforms. Other applications include the comparison of temporally sampled data or data derived from different tissues within the host. Given that novel next generation sequencing platforms and chemistries are being developed, ease of comparison of data from different sources is timely. Our software permits the efficient analysis of multiple such data sets within one integrated framework. We demonstrate its usage by comparing extremely large data sets of influenza H1N1 samples sequenced on both the 454 Life Sciences and Illumina platforms. Previously we have demonstrated how phylogenetic trees can be inferred from combined time points of viral data, permitting the tracking of the emergence of distinct viral lineages associated with HIV-1 drug resistance forms [3,35].

To determine the importance of characterizing the extent of platform induced variation in this study we compared the entropy present within the read data derived from the 12 H1N1/09 samples to that of a sample of data from GenBank (Figure 4C). We observed that the median entropies obtained from reads pooled according to platform were higher than those obtained from the GenBank data, thus, highlighting the importance of quantifying how much of this variation was caused by platform error, prior to the detection of genuine variants. In order to quantify this we considered any differences in variation between the data from each of the platforms as being platform dependent (Figure 5). Following the calculation of the per site ratios between variant and consensus frequencies, it was observed that for insertions and deletions the ratios within the 454 data were significantly higher than their Illumina counterparts (Figure 6A). The higher ratios were strongly associated with hps^+ ^regions (Figure 6B), a characteristic that has been previously observed, for example, within a study designed to characterize platform error using the reverse transcriptase gene of HIV-1 [7]. This result highlights the suitability of the Illumina technology for viral data sets, which is often prone to high levels of biologically relevant indels.

While mapping of low frequency variation at sites on the template to locations within the underlying reads it was observed that within the 454 data, with the exception of the read starts, the frequencies follow a random expectation (Figure 7A, C and 7E). This is unsurprising given the observation that the majority of platform-induced variation is dependent on the location of sites in relation to hps^+ ^regions (Figure 6B), as defined by the genome. Since these regions are located randomly in relation to the read, platform dependent variation would be expected to appear randomly with respect to read length. The major implication of this is that that error occurring at particular positions on a genome may be replicated across multiple independent samples, as has been previously observed [27]. It also suggests, that within the 454 data the genomic positioning of hps^+ ^sites can be used to accurately predict where this error is likely to occur, and that steps can be taken to reduce its effects on data analysis, such as the removal of insertions within reads that fall with hps^+ ^regions and that are associated with low quality scores. Conversely within the data obtained from the Illumina platform the distributions of indel error in relation to read location does not fall consistently within the random expectation (Figure 7B, D and 7F). For this data hps^+ ^regions were not observed to influence location. Combined, this suggests that read location plays an influential role in the introduction of such error, although the levels present, especially given the far higher coverage, are extremely low.

For mismatches, ratios between consensus and variant frequencies were observed to be relatively similar on both platforms, although as a result of higher coverage many more sites within the Illumina data harbored low-level change (Figure 8A and 8B). To demonstrate the applicability of our software to data sets derived from different sources the topology generated by the number of variable sites cross-validated using both platforms, in relation to the quality of the nucleotides present was plotted (Figure 9). The topology follows the general trend of high non-consensus and quality score values lead to few polymorphic sites, low non-consensus and quality score thresholds lead to many polymorphic sites. Of particular interest, however, is that between the 55^th ^and 56^th ^quality percentiles there is a consistent decrease in the number of variable sites identified, suggesting a possible cutoff value for this parameter. Interestingly, quality alone does not appear to be sufficient for accurately identifying the presence of low-level biological variation as, without cross-validation using the Illumina platform, the number of sites identified within each sample is consistently higher (Figure 10). In the latter the median number of variable sites across all 12 samples is 1947, which is almost three times higher than when cross-platform validation is performed. Reflecting this uncertainty is the development of many probabilistic methods that attempt to improve the reliability of identifying low-level variation at sites within data obtained from a single platform [36-39]. Our frameworks ability to store temporally sampled data provides an opportunity to derive a set of priors characterizing the expected variation within that individual. Combined with the error rates described here and in conjunction with read quality scores this forms the bases for our first future update which involves filtering platform dependent variation from temporally sampled read data using a Bayesian approach.

## Conclusion

We have provided software, Segminator II, which can be used for the processing of temporally, spatially or otherwise linked viral data obtained from next generation sequencing platforms. In a demonstration of the usability of our software we have also quantified the amount of platform dependent error that is present within data generated on both the 454 and Illumina platforms and, thus, highlighted the need for care when calling low frequency variants using a single platform. Given that next generation data is increasingly important in the analysis of drug-resistance and vaccine trials, this software will be useful to the retroviral research community.

## Availability and requirements

*Project name: *Segminator II

*Project home page: *http://www.bioinf.manchester.ac.uk/segminator/

*Operating system: *e.g. Platform independent

*Programming language: *Java

*Other requirements: *Java 1.6 or higher

*License: *GNU Lesser GPL

*Any restrictions to use by non-academics: *license needed

## Competing interests

The authors declare that they have no competing interests.

## Authors' contributions

Conceived and designed the project: DLR, AR and JA. Performed the sequencing: PK, GB and SW. Analyzed the data: JA, DLR and AR. Implemented the software: JA. Wrote the paper: JA, with input from DLR, AR and PK. All authors commented on and approved the final version of the manuscript.
